# Predictive Model and Mortality Risk Score during Admission for Ischaemic Stroke with Conservative Treatment

**DOI:** 10.3390/ijerph19063182

**Published:** 2022-03-08

**Authors:** María Carmen Lea-Pereira, Laura Amaya-Pascasio, Patricia Martínez-Sánchez, María del Mar Rodríguez Salvador, José Galván-Espinosa, Luis Téllez-Ramírez, Fernando Reche-Lorite, María-José Sánchez, Juan Manuel García-Torrecillas

**Affiliations:** 1 Internal Medicine Department, Hospital de Poniente, El Ejido, 04700 Almería, Spain; mariadelcarmen.lea@ephpo.es; 2 Department of Neurology and Stroke Unit, Hospital Universitario Torrecárdenas, 04009 Almería, Spain; laura.amaya.pascasio.sspa@juntadeandalucia.es (L.A.-P.); patricia.martinez.sanchez.sspa@juntadeandalucia.es (P.M.-S.); 3 Nurse in Almería Primary Care District, 04009 Almería, Spain; mariam.rodriguez.salvador.sspa@juntadeandalucia.es; 4 Alejandro Otero Research Foundation (FIBAO), Hospital Universitario Torrecárdenas, 04009 Almería, Spain; jgalvan@fibao.es; 5 Biomedical Research Unit, Hospital Universitario Torrecárdenas, 04009 Almería, Spain; ltr385@inlumine.ual.es; 6 Department of Mathematics, University of Almería, 04120 Almería, Spain; freche@ual.es; 7 Escuela Andaluza de Salud Pública, 18011 Granada, Spain; mariajose.sanchez.easp@juntadeandalucia.es; 8 Instituto de Investigación Biomédica Ibs. Granada, 18012 Granada, Spain; 9 Centro de Investigación Biomédica en Red de Epidemiología y Salud Pública (CIBERESP), 28029 Madrid, Spain; 10 Department of Preventive Medicine and Public Health, University of Granada, 18071 Granada, Spain; 11 Department of Emergency Medicine, Hospital Universitario Torrecárdenas, 04009 Almería, Spain

**Keywords:** predictive model, risk score, mortality, stroke, vascular neurology

## Abstract

Background: Stroke is the second cause of mortality worldwide and the first in women. The aim of this study is to develop a predictive model to estimate the risk of mortality in the admission of patients who have not received reperfusion treatment. Methods: A retrospective cohort study was conducted of a clinical–administrative database, reflecting all cases of non-reperfused ischaemic stroke admitted to Spanish hospitals during the period 2008–2012. A predictive model based on logistic regression was developed on a training cohort and later validated by the “hold-out” method. Complementary machine learning techniques were also explored. Results: The resulting model had the following nine variables, all readily obtainable during initial care. Age (OR 1.069), female sex (OR 1.202), readmission (OR 2.008), hypertension (OR 0.726), diabetes (OR 1.105), atrial fibrillation (OR 1.537), dyslipidaemia (0.638), heart failure (OR 1.518) and neurological symptoms suggestive of posterior fossa involvement (OR 2.639). The predictability was moderate (AUC 0.742, 95% CI: 0.737–0.747), with good visual calibration; Pearson’s chi-square test revealed non-significant calibration. An easily consulted risk score was prepared. Conclusions: It is possible to create a predictive model of mortality for patients with ischaemic stroke from which important advances can be made towards optimising the quality and efficiency of care. The model results are available within a few minutes of admission and would provide a valuable complementary resource for the neurologist.

## 1. Introduction

Ischaemic stroke is the second cause of mortality in Spain in the general population and the first in women [1]. It is also the second cause of mortality worldwide and the third most common in industrialised countries [2,3]. The prevalence of this major public health problem puts significant strain on health system resources.

A large proportion of strokes (approximately 62%) are attributable to ischaemic processes, many of which are not treated with reperfusion treatment (pharmacological or mechanical) because they do not meet the strict criteria for its application [4,5]. Determining the proportion of patients undergoing reperfusion is a complex task. Among other factors, it depends on the time of onset of the stroke, the priorities and capacities of the health system and the emergency management elements available in each case. A recent study using data from 44 European countries reported a mean reperfusion rate with thrombolysis of 72.5 cases per 1000 stroke episodes; with mechanical thrombectomy, this rate fell to 19.3 cases per 1000 episodes [5,6]. Although these figures are open to improvement, it is apparent that the period from the onset of the stroke to the administration of the fibrinolytic therapy or thrombectomy provides a window of opportunity for successful treatment. Thus, during the first three hours following the onset of this condition, the application of fibrinolytics achieves a marked reduction in disability in 25% of patients. Mechanical thrombectomy, which is normally only available in specialised centres and presents a heterogeneous international distribution, also increases survival rates and drastically decreases sequelae [7,8,9,10]. However, a high proportion of strokes are not suitable for any type of reperfusion, and it is these on which our study is focused.

Various risk factors have been detected for the occurrence of an ischaemic event and for the probability of death or major sequelae [11,12]. Among other studies in this respect, Smith et al. [13] proposed a predictive model for hospital mortality following ischaemic or haemorrhagic stroke. This model had a moderate–high discriminant capacity. Other studies have also reported satisfactory predictive models to assess the risk of mortality in acute stroke [14,15,16,17,18].

In Spain, studies have been undertaken to establish the determinant factors of mortality due to ischaemic stroke, based on an analysis of hospital records. However, to our knowledge, none have proposed a predictive model based on the national database that provides the basic information obtained for each hospital admission episode (the Spanish Minimum Basic Data Set, MBDS). Analysis of data from this source would allow us to estimate the probability of mortality during the hospitalisation for acute stroke when reperfusion treatment is not provided. However, these administrative data are not amenable to analysis, and it can be a complex task to determine the severity of the stroke exclusively from the information contained in this database [19].

In addition, the aforementioned characteristics of stroke have recently led to an increasingly frequent use of machine learning (ML) or deep learning (DL) techniques. Recently, attempts have been made to predict stroke mortality using principal component analysis methods scaled to neural networks with very good predictive results [20]. In addition to stroke, other pathologies such as heart failure and sepsis are being studied using this methodology. In the case of heart failure, there are promising ML studies that validate and increase the performance of classical statistical models [21]. In the study of mortality due to sepsis, the use of convolutional neural networks is gaining ground [22].

When a stroke patient is admitted to the emergency room, the time elapsed since the event, the therapeutic measures adopted and the training of the care team are of decisive importance in reducing in-hospital mortality.

Subsidiary data compiled on admission or during the first hours of the patient’s hospital stay can be incorporated into a predictive model that can be used to estimate the probability of mortality during admission. The successful development and application of such a model would have important benefits for hospital procedures and efficacy.

On admission following a stroke, patients are rigorously evaluated by specialised personnel, who assess not only the degree of severity but also the time elapsed from the onset of symptoms to the provision of initial care. Many patients are not considered candidates for reperfusion treatment because the window for treatment has closed, because they present contraindications or because there is a high risk of haemorrhagic transformation, among other reasons. For these patients, an individualised prognostic study is required.

In view of these considerations, we aim to construct a predictive model to identify patients at greatest risk of dying during admission for ischaemic stroke when they are not eligible for reperfusion treatment. Such a model would make it possible to optimise clinical actions and improve treatment outcomes for this substantial group of patients. In a complementary analysis, we also assess the performance of ML–DL techniques.

## 2. Materials and Methods

### 2.1. Study Design and Data Source

This analytical observational study considers a cohort representing all the episodes of hospitalisation in Spain for patients with ischaemic stroke and who did not receive reperfusion therapy (diagnostic group 14 in the Spanish classification system, GRD), during the period 2008–2012. It was assumed that the attribution of cases to DRG-14 was correct in all cases.

The source of information was the Minimum Basic Data Set (MBDS) compiled by the Ministry of Health, Consumer Affairs and Social Welfare. This database contains both treatment and administrative information. The system originated in the USA and was later adopted by the European Economic Community. In Spain, the MBDS has been of mandatory implementation since 1987.

The unit of statistical analysis considered was the hospitalisation episode and not the individual; in total, 186,245 hospitalisation episodes classed as GRD-14 were analysed. Each group within this system is homogeneous by resource consumption but not in terms of disease severity. Accordingly, the processes can be grouped to enable comparability between different health regions and geographical areas.

To facilitate subsequent internal validation, the cohort was randomly segmented into two subsamples, a training set (80% of the episodes) and a test set (20% of the episodes). These subcohorts were established using a blind process of simple random sampling, and all statistical studies except the internal validation process were performed on the training set.

### 2.2. Variables

In all our statistical analyses, the main variable was in-hospital mortality, that is, death during hospitalisation due to an ischaemic stroke when no reperfusion treatment had been received. This variable was used to develop the intermediate and final models, as well as during the validation process.

The remaining variables were taken as independent or predictor variables and were composed of two main groups: the sociodemographic covariates of age, sex and geographic origin; and the comorbidities, mainly ischaemic heart disease, chronic bronchopneumopathy, renal and/or respiratory insufficiency, dyslipidaemia, arterial hypertension, diabetes, obesity, valvular heart disease, anaemia, arrhythmias—especially atrial fibrillation—and pneumonia.

In addition to the above, a specific analysis was performed of case management variables and those related to hospital admission characteristics. The latter included the number of diagnoses on discharge (NDD), as a proxy for the level of comorbidities, and the number of procedures at discharge (NPD), as a proxy for the diagnostic–therapeutic effort received. Other key variables were the length of stay and readmission for the same reason within one month of discharge.

The definition of readmission deserves special attention. The approximation to the number of patients and readmissions was made by identifying the same patient for a hospital and for a specific year, based on the history number, hospital code, date of birth and sex. This allowed us to identify patients treated more than once in the same hospital.

The database was subjected to a moderate screening procedure for outliers regarding the length of stay, using the formula T2 = Q3 + 1.5 (Q3 − Q1), where Q is the quartile and T2 is the length of stay for DRG-14 above which values are considered extreme. In the present case, stays exceeding 21 days were considered atypical.

### 2.3. Statistical Analysis

The statistical analysis was carried out on the training cohort except for the final phase, in which the results were validated on the test cohort using the hold-out method. The entire analytical procedure was performed using SPSS v.21 (IBM Corp., Armonk, NY, USA and Stata v.14 software (StataCorp LLC, College Station, TX, USA).

The initial phase of the analysis consisted of a descriptive study of the variables of interest (sex, age, NDD, NPD, readmission, mortality and comorbidities). The qualitative variables are expressed as percentages and frequency distributions, and the quantitative ones as means and standard deviations.

A bivariate analysis was performed to detect associations between mortality and each of the predictors considered. These associations are expressed as unadjusted odds ratios (ORu), accompanied by the corresponding 95% confidence interval and statistical significance.

Finally, a binary logistic regression model was developed for the dependent variable “mortality”, using the manual introduction method to force the inclusion of significant variables in the bivariate analysis, together with other appropriate variables, according to the literature. The variables were manually withdrawn from the model according to their degree of contribution to its overall performance, prioritising the withdrawal of those that were not present at the moment of hospital admission or very shortly afterwards.

The model was evaluated for its discriminant capacity using the area under the curve (AUC) or C-statistic. The calibration was evaluated by visual comparison of the risk deciles obtained using the Hosmer−Lemeshow test, since the level of significance did not provide a suitable measure, due to the large sample size; in this case, therefore, visual interpretation provides more information. Pearson’s chi-square goodness-of-fit test was also used, as it is more appropriate when a large sample size is considered [23].

Finally, the model developed on the training cohort was applied to the test cohort, by the hold-out method, to establish internal validity. The AUC values obtained for each cohort were compared using the method described by DeLong et al. [24].

To facilitate an immediate estimation of the risk of mortality and to enable rapid access to the results during treatment for patients with stroke, we created a smartphone app and a web equivalent (URL: https://calculadora-ictus.firebaseapp.com/, accessed on 1 June 2021). A mobile application for smartphones was designed using the free framework “Ionic” [25] and Visual Studio Code. In order to achieve greater accessibility from any location with Internet access from this application, a progressive web application (PWA) was created, which is accessible from the aforementioned URL.

From the above procedures, a risk score for mortality was derived, using the coefficients of the logistic regression model, as described by Sullivan et al. [26]. In this method, each coefficient is divided by the lowest value, and the score thus obtained is rounded to the nearest integer, which is taken as the weight assigned to each factor in the final score. For each patient included in the study, we calculated the total score according to the presence or absence of each factor. The score obtained for each patient was in the range from −12 to 61 points.

A complementary analysis of the database was conducted using deep learning (DL) techniques. The most relevant variables for the model were selected using random forest (RF) [27], and the permutations test was used as a further correction mechanism [28,29]. After selecting the variables with the highest predictive capacity, a multilayer perceptron neural network was constructed, and the performances of the network and the random forest procedure per se were obtained.

## 3. Results

The final analysis consisted of 186,245 episodes of hospitalisation for non-reperfused stroke. Of these episodes, 80% (*n* = 148,891) were taken as the training cohort and 20% (*n* = 37,354) as the test cohort. All statistical studies except the internal validation process were performed on the former.

The patients were mainly elderly (mean age 73.92 ± 12.53 years) and the mean duration of hospital stay was 7.54 days. Just over half (53.30%) were male and 4.8% were in readmission due to ictal symptoms. In-hospital mortality during the initial admission was 6.8%. Table 1 lists the main comorbidities presented and the other variables analysed.

**Figure 1:**
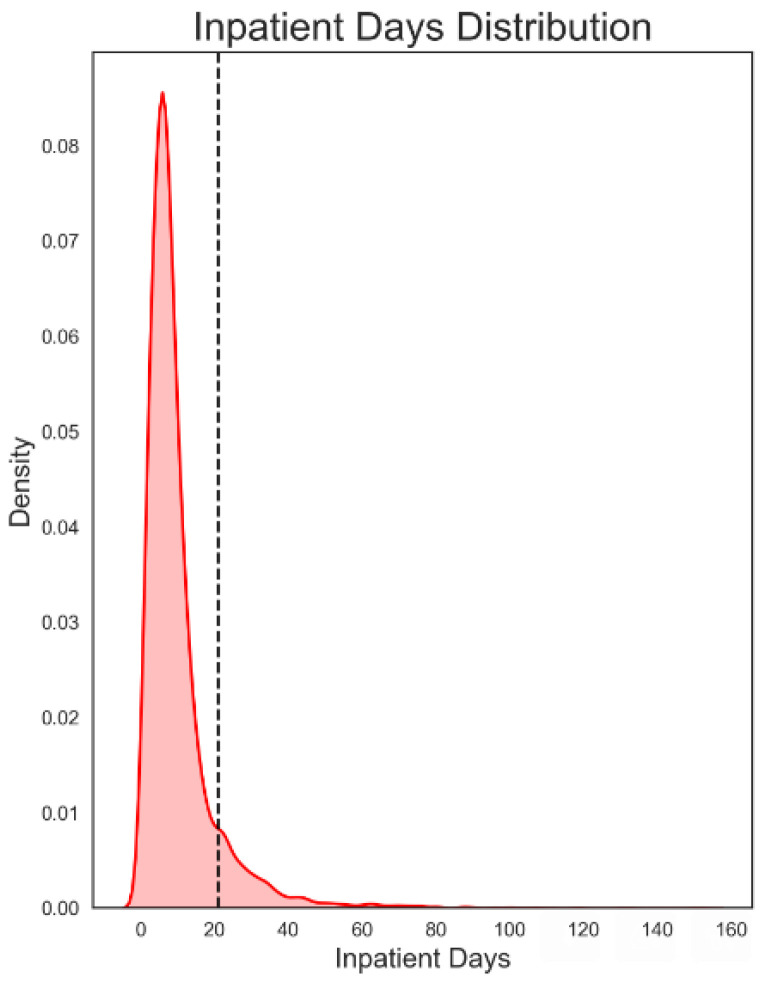
Length of stay. Cutoff point for outliers. Cutoff point for outliers: 21 days.

The proportion of outliers was 6.11% when a stay of more than 21 days was considered an atypical stay (Figure 1). After removing the outliers, the global database was composed of 186,245 episodes of hospitalisation for non-reperfused stroke.

The bivariate analysis (Table 2) revealed significant differences in several of the variables. Thus, positive associations with mortality were obtained for female sex (ORu 1.792, 95% CI: 1.720–1.787), readmission (OR 2.324, 95% CI 2.165–2.493) and among comorbidities, COPD (OR 1.188, 95% CI: 1.101–1.282), renal failure (OR 1.664, 95% CI: 1.551–1.786), atrial fibrillation (OR 2.414, 95% CI: 2.316–2.516) and ischaemic heart disease (OR 1.324, 95% CI: 1.217–1.441). On the contrary, both dyslipidaemia (OR 0.489, 95% CI: 0.466–0.514) and hypertension (OR 0.792, 95% CI: 0.760–0.826) were negatively associated with the risk of death during the admission.

The final logistic model built on the training cohort was composed of ten variables and provided an AUC of 0.742 (95% CI: 0.737–0.747). This model was also applied to the test cohort, and both presented a moderate–high and very similar discriminant capacity (Table 3).

**Figure 2:**
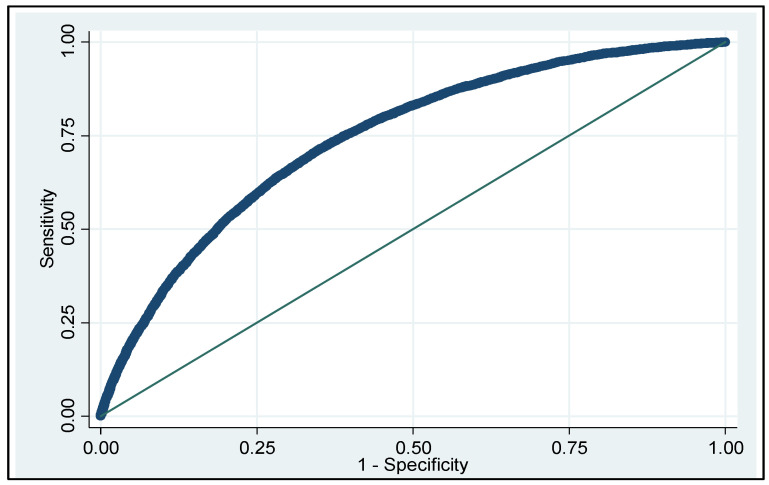
ROC curve of the mortality predictor model applied to the training cohort. AUC 0.742, 95% CI: 0.737–0.747.

The logistic model built from the training cohort was composed of ten variables and presented a moderate–high discriminant capacity, with an AUC of 0.742 (Table 3, Figure 2).

**Figure 3:**
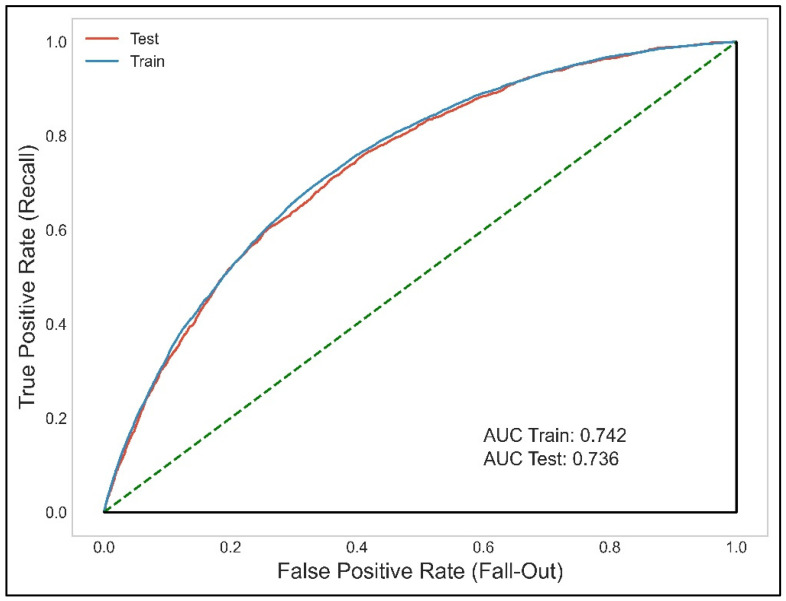
ROC curves for the training and test cohorts. AUC Train: 0.742, 95% CI: 0.737–0.74; AUC Test: 0.736, 95% CI: 0.727–0.746. AUC Train − AUC Test = 0.006, CI difference of the means 0.002–0.005.

Application of the model to the test cohort (to verify its internal validity) produced an AUC of 0.736, 95% CI 0.727–0.746 (Figure 3). The difference in the AUC was 0.006 units and the DeLong test was significant (*p* < 0.001) despite the minimal differences in relation to the large sample size. The model met the classical internal validation criterion, i.e., a decrease in the C-statistic of no more than one-tenth of a unit.

**Figure 4:**
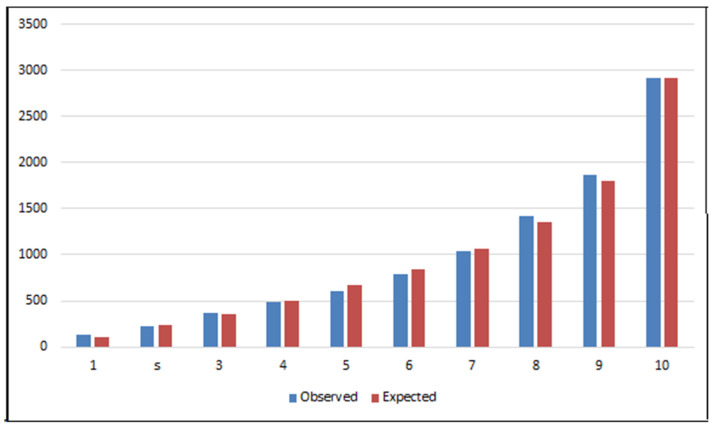
Graphical representation of the risk deciles by the Hosmer–Lemeshow test. Hosmer–Lemeshow test values: χ2 = 26.094, *p* = 0.01.

The calibration was also evaluated by the Hosmer–Lemeshow test and through the visual representation of the risk deciles (Figure 4). The Pearson test [30], which is highly recommended for large sample sizes, was also applied. This test showed there were no significant differences between the values observed and those predicted by the model (Pearson χ2 ungrouped, *p* = 0.176); the visual interpretation of the observed and expected deciles is consistent with this result.

**Figure 5:**
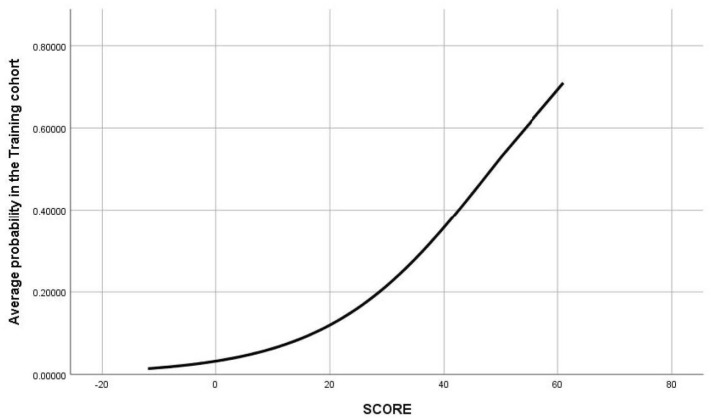
Relation between the risk score and the risk of death.

The risk score produced values in the range of −12 to 61 points. Its graphical representation facilitates a rapid estimate of the relation between the score on the probability curve and the risk of death (Table 4 and Figure 5).

**Figure 6:**
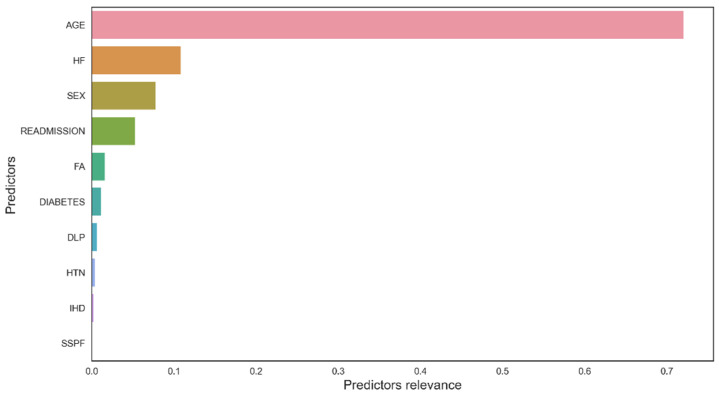
Random forests. Relevance of predictors for mortality. HF: heart failure; FA: atrial fibrillation; DLP: dyslipidaemia; HTN: arterial hypertension; IHD: ischaemic heart disease; SSPF: Symptoms suggestive of posterior fossa infarction.

**Figure 7:**
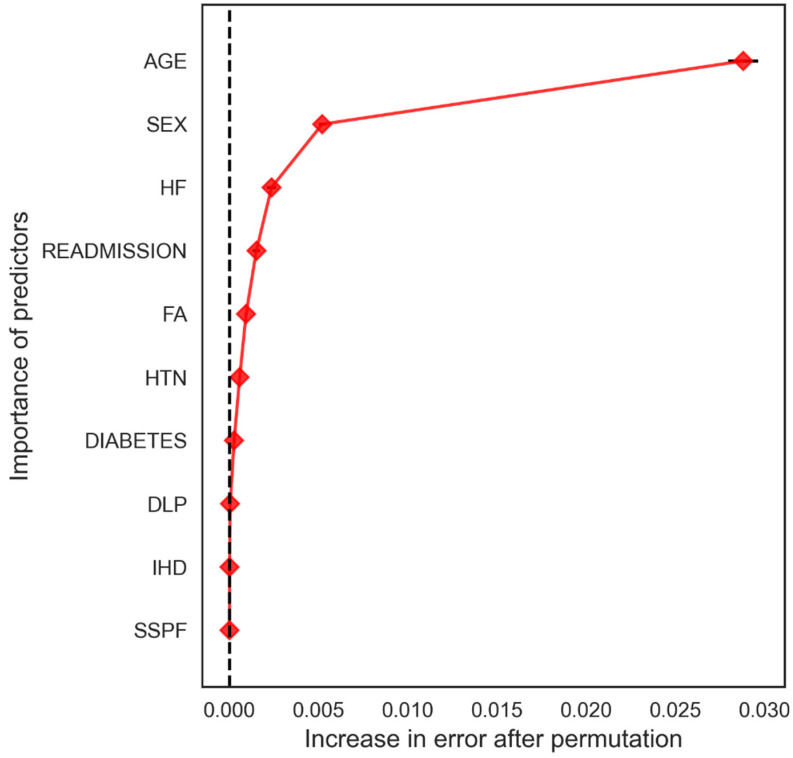
Permutation test. Correction of predictors after permutation.

From the perspective of DL, a random forest analysis provided the variables with the highest weights as possible predictors of mortality (Figure 6), with age, heart failure, readmission and female sex, among others, standing out. The permutation test reduced the overestimation of the RF (Figure 7) although the same variables were maintained.

Using the variables selected from the previous tests as predictors, a multilayer perceptron neural network presented an AUC of 0.651. Table 5 shows the comparative values of the different metrics obtained for the different methodologies used.

## 4. Discussion

### 4.1. Findings

In this study, we analyse information obtained from clinical–administrative databases managed by the Spanish Ministry of Health, with respect to the period 2008–2012. These data were used to develop a predictive model based on logistic regression with a moderate–high capacity to estimate the risk of mortality in patients hospitalised for an ischaemic stroke when neither thrombolysis nor thrombectomy have been performed.

The model obtained provides an accurate visual calibration in terms of the concordance between the observed values and the mortality predicted according to the risk deciles obtained by the Hosmer–Lemeshow method. To streamline the risk estimation procedure, we also developed an auxiliary web application that allows the exact risk to be estimated almost immediately [31].

As the final outcome, we obtained a score to represent the mortality risk of each patient. It can be derived quickly and easily, and we believe it will be of great use to the clinician. It can be accompanied by a graphical representation that is equally fast and easy to use, either in combination with the results of the logistic model or independently.

On the other hand, the DL-based approach showed that modern data science methodologies do not always provide better results than more traditional statistical techniques. In this sense, logistic regression showed a predictive performance equal or superior to RF and neural network methodologies.

The use of techniques such as ML or DL to tackle this type of problem is growing every day, but in the studies consulted, there is one element that tends to be constant, and that is the use of databases that are more specific and oriented towards specific pathologies than the one we have used [20,22].

One of the frequent problems when working with databases in which the outcome variable represents a very small proportion is the imbalance of the data. There is a tendency towards asymmetry and imbalance in the classification tables that makes it necessary to use metrics other than the AUC-ROC, such as accuracy, precision and recall metrics. All of these are displayed in Table 5 and denote an acceptable behaviour of the model despite the difficulties imposed by the dataset.

### 4.2. Comparison with Previous Studies

Numerous prediction models and mortality scores for stroke patients have been presented, but to our knowledge, none in Spain based on the MBDS or any other clinical-administrative database using such a high volume of cases as in the present study. Instead, reference is frequently made to clinical records, using only small-to-moderate sample sizes and, in most cases, without generating a complete model. On the other hand, interesting studies based on artificial intelligence, using the random forest method, have been made to predict mortality risk. However, the theoretical basis, the details of the method applied and the combined use of clinical, neuroimaging and biochemical markers substantially distance these studies from the approach we describe [32]. Moreover, the model proposed in this paper can be built to obtain a very rapid estimate, during the initial assessment of the patient, thus enhancing care efficiency.

Numerous studies have been conducted in other countries, using various sources of information to develop and validate mortality risk scores, but most of them present significant differences from our work, especially as concerns the medium- to long-term estimation of mortality risk [13,15,16,17,18,19,33].

The PREMISE score, for predicting early mortality due to stroke, was developed from a prospective register with data from 38 stroke units in Austria. This score has been validated internally and externally and provides optimal results [16,34]. From a multi-variate model, the authors derived a score that performed well in terms of discriminant capacity (AUC 0.879) and visual calibration. The information source used in this study was a group of stroke units that supplied data to a specific national register. This source, hence, was much more precise than our own (a national clinical–administrative database that presents a certain lack of exhaustiveness, as it is not designed specifically for recording details of patients who have suffered a stroke). Clinical severity instruments such as the NIH Stroke Scale/Score (NIHSS), although they make comparability more complex, also enhance predictive capacity.

Three important scores have been developed to estimate short- and medium-term mortality. Two of these (providing 30-day estimates) were produced by the Canadian Stroke Network, and the third considered in-hospital mortality in a way similar to the approach we describe (Smith et al., 2010) [13]. In the latter work, a risk score method was devised to estimate in-hospital mortality, based on a study of 274,988 patients treated in 1036 U.S. hospitals. The study data were obtained from the “Get with the Guidelines—Stroke Program” [35,36]. Using this information, the authors developed referral and validation cohorts, alternately including and omitting the severity scale of the NIHSS. This procedure generated two risk models: the one excluding the NIHSS had a discriminant capacity of 0.72, and the one including it had a discriminant capacity of 0.85. Although the latter was clearly better, in both cases, discrimination was good, as was the visual plot calibration.

The results obtained in the present study reveal a discriminant capacity (based on the C-statistic) similar to that of Smith et al. when the NIHSS severity scale was not used. In common with these authors, we not only developed a model that excluded this key variable but also created one that both incorporates estimates of in-hospital mortality and uses only the variables present at the time of admission. This feature is extremely important, since knowing the patient’s risk profile in the first few minutes of evaluation facilitates the use of personalised medicine and enables clinical circuits and schedules to be organised for optimal effectiveness and efficiency.

Notably, therefore, our model obtains a similar discriminant capacity to that offered by the one proposed by Smith et al., in both cases excluding the NIHSS variable. This is a useful achievement because our model is based on a clinical–administrative information source, which is inevitably less exhaustive than the database used in the earlier study [37]. The visual representation of the risk deciles (observed vs. expected) in the Hosmer–Lemeshow (HL) test shows our calibration data to be acceptable. However, the HL test is based on a chi-square test, which with such large sample sizes is usually non-significant. To avoid this limitation, we applied the Pearson χ2 test, implemented by Stata software [38], which confirmed the absence of significant differences in our calibration analysis, as we hoped.

Studies in this respect have also been conducted in non-industrialised countries, for example by Gondar et al. [33]. These authors analysed medical records from Ethiopia, producing a predictive model to assess the risk of in-hospital mortality due to ischaemic stroke. However, the sample size was only modest, and the multivariate analysis performed was unable to achieve an optimal model based on logistic regression. However, a subanalysis based on Cox proportional hazards showed that intercurrent infections and impaired renal function were associated with higher mortality while, as in our study, dyslipidaemia was associated with lower mortality.

Finally, a recent study conducted in Canada proposed a risk score to classify stroke severity, basing their analysis on administrative data [19]. The discriminant capacity of this score was evaluated according to the inclusion of certain critical variables, producing an AUC value of 0.82 when the Canadian Neurological Scale (CNS) with real observed data was included. This value fell to 0.76 when the CNS was used with the data estimated by the model and further still when the CNS was excluded (C = 0.69). This model has the undoubted advantage of its inclusion of critical variables (via the CNS) in the assessment of stroke mortality risk. Nevertheless, our own model, despite lacking these critical variables, obtained results similar to those of the intermediate model above, derived from the CNS.

In conclusion, having compared the outcomes of the present study with those reported in the literature in this regard, we believe our work is consistent in methodology and results with most international studies in this field and achieves results similar to those of studies that use only general clinical and administrative variables and not those of a critical nature in patients with stroke.

### 4.3. Strengths of the Study

We present a model for estimating the risk of mortality in patients admitted for an acute non-reperfused stroke. This model is composed of variables obtained from clinical–administrative databases and provides moderate–high discriminant capacity and good calibration. It is the first such model to be based on the Spanish Minimum Basic Data Set that considers mortality, and, moreover, it is accompanied by a smartphone app that facilitates immediate estimation of the mortality risk.

Another major strength of this study is the large sample size considered and the long duration of the data collection period (five years). In addition, it takes into account all patients admitted for stroke, nationwide, during this period.

Prior to our investigation, many such risk scores and prediction models have been developed to address the same problem, but most attempt to predict mortality at different times after hospital discharge. Furthermore, the majority are constructed using information from real-life cases, not from clinical–administrative databases.

In most of these previous studies, the patients included in the analysis were treated in hospital stroke units or, to a lesser extent, conventional neurology wards. In consequence, little research attention has been paid to the profile of patients addressed in the present study [16,17,34]. Moreover, some studies examined only non-reperfused ischaemic strokes while others addressed haemorrhagic strokes and ICU treatment in separate analyses [15]. It is important to note that stroke units make greater use of specialist procedures and therapeutic effort than is the case with patients managed conservatively, as is the case of those examined in our study [38]. Only Aylin et al. [39] used data from a database comparable to the Spanish MDBS (in their case, from the British NHS) but this analysis was applied to non-cerebral vascular pathologies. In relation to the latter, therefore, our prediction and score model can be considered totally novel. Furthermore, the creation of a web tool, accessible from any device with an Internet connection, together with the provision of a smartphone application, enable the physician to resolve a complex logistic equation almost immediately, taking into account only variables that can readily be evaluated in the first moments of patient care.

Finally, a notable strength of the study is the model’s “explainability”. The model presented is easily interpretable, and the approach using logistic regression is optimal from the point of view of explainability, as this method makes it possible to understand the way in which the predictions are made with relative simplicity. The model is also very easy to implement (in a mobile application, on the web, etc.), and it can be easily modified and re-adapted after external validation processes, using new coefficients with significant efficiency.

### 4.4. Potential Limitations

A potentially significant limitation of the present study is the absence of data from the NIHSS scale in our source of information, which makes it much more difficult to raise the discriminant capacity of the model. Nevertheless, moderate, efficient AUC levels are achieved for use in clinical practice.

In addition, the databases consulted do not contain sufficient information to determine the severity of the stroke upon admission. For example, in our study, it was not possible to obtain variables enabling us to reproduce or estimate the NIHSS or the CNS. This inevitably reduces the precision obtained, compared to studies that do consider these critical variables. The Spanish clinical–administrative database is not pathology specific but is designed to encode any pathological process.

The classification used in this study (ICD 9MC) predates the ICD10 version currently in use; the clinical and outcome profile of patients with reperfusion criteria (thrombectomy or thrombolysis) has changed over the years, something that is not observed for patients who are managed conservatively. In fact, increasing the time available from stroke onset for the possible application of new therapies has been the main change in stroke management.

In the present study, we have assumed a correct attribution of episodes to DRG 14; undoubtedly there is a source of variability in coding that may make this a priori assumption a limitation to some extent. Moreover, the origin of the data is very heterogeneous and difficult to integrate, as it combines information from a large number of regional databases. In relation to these characteristics of the ICD version used, no attempt has been made to validate the obtained model outside the country or to other classifications, which would have placed a significant bias on the study. Finally, our study may be subject to underreporting bias, a well-known limitation of this type of information source [40].

### 4.5. Implications

The study we describe proposes a method to determine the stratification of mortality risk in a patient being treated in the emergency room (with no reperfusion) after undergoing an acute ischaemic stroke. This parameter is of fundamental importance in establishing the type and urgency of healthcare to be applied.

Personalising this risk allows clinicians to optimise the pathways provided and the complementary tests performed, ensuring that critical times are not exceeded. By establishing maximum delay times before consultation with the neurology specialist and time limits for performing imaging tests or initial care, and ultimately by promoting personalised medicine targeting the specific risk presented, hospitals can optimise the care provided for patients with this pathology. Furthermore, these measures would be valid not only for urgent care but also for more routine decision-making in the hospitalisation ward.

The model proposed is not intended to replace, under any circumstances, the opinion of the expert neurologist. However, we believe it will be a valuable resource when used in conjunction with clinical scales and specialist opinion.

## 5. Conclusions

We propose a practical means of obtaining a predictive model of the risk of mortality during the admission of patients with non-reperfused stroke. The use of such a model would have beneficial clinical and healthcare repercussions and contribute to optimising care for this group of patients.

Finally, we emphasise that, although the discriminant capacity of this model is similar to that offered in related work, our model and risk score are based exclusively on variables that can be determined just a few minutes after admission, with evident advantages for patient care.

## Figures and Tables

**Table 1:** Descriptive variables.

VARIABLE		
**Quantitative, mean ± sd**	Age	73.92 ± 12.53 (years)
	Stay	7.54 ± 4.54 (days)
	NDD	6.92 ± 2.95 (diagnostics)
	NPD	3.27 ± 2.45 (procedures)
Qualitative, *n* (%)	Male sex	79,412 (53.30)
	Re-admission	7140 (4.80)
	Mortality	10,141 (6.80)
	COPD	9898 (6.60)
	Ischaemic heart disease	7181 (4.80)
	Arterial hypertension	95,822 (64.4)
	Obesity	9358 (6.30)
	Renal insufficiency	9035 (6.10)
	Anaemia	7840 (5.30)
	Atrial fibrillation	37,050 (24.9)
	Diabetes	46,241 (31.10)
	Dyslipidaemia	52,857 (35.70)
	Heart failure	11,141 (7.50)
	Basilar artery stenosis	660 (4.00)

NDD: number of diagnoses on discharge; NDP: number of procedures on discharge; COPD: chronic obstructive pulmonary disease.

**Table 2:** Bivariate study. Mortality-associated factors in the training cohort.

		**Total**		**Death**				
		***n***	**%**	***n***	%*	**ORu**	**95% CI**	***p***
Sex	Male	79,412	53.3	4045	5.1	1		
	Female	69,473	46.7	6096	8.8	1.792	1.720–1.787	<0.0001
Age	24–34	853	0.6	8	0.9	1		
	35–44	2791	1.9	37	1.3	1.419	0.658–3.059	0.372
	45–54	9233	6.2	124	1.3	1.438	0.701–2.949	0.322
	55–64	18,857	12.7	381	2	2.178	1.078–4.402	0.030
	65–74	33,122	22.3	1196	3.6	3.957	1.968–7.957	<0.001
	75–84	54,662	36.7	3856	7.1	8.017	3.993–16.095	<0.001
	>84	29,373	19.7	4539	15.5	19.305	9.616–38.758	<0.001
Year	2008	29,509	19.8	2104	7.1	1		
	2009	29,577	19.8	2076	7.0	0.983	0.923–1.047	0.559
	2010	30,019	20.2	2059	6.9	0.959	0.901–1.022	0.195
	2011	29,885	20.1	1966	6.6	0.917	0.861–0.978	0.008
	2012	29,901	20.1	1936	6.5	0.902	0.846–0.961	0.002
NDD	0–3	17,460	11.7	1414	8.1	1		
	4–7	72,890	49.0	4686	6.4	0.78	0.733–0.829	<0.0001
	8–11	46,083	31.0	3137	6.8	0.829	0.766–0.885	<0.0001
	≥12	12,458	8.4	904	7.3	0.888	0.814–0.968	0.007
NPD	0–3	87,960	59.1	7909	9.0	1		
	4–7	52,485	35.3	1930	3.7	0.386	0.367–0.407	<0.0001
	8–11	7385	5	255	3.5	0.362	0.319–0.411	<0.0001
	12–15	935	0.6	41	4.4	0.464	0.339–0.635	<0.0001
	≥16	126	0.1	6	4.8	0.506	0.223–1.149	0.104
COPD	No	138,929	93.3	9355	6.7	1		
	Yes	9898	6.6	782	7.9	1.188	1.101–1.282	<0.0001
Arterialhypertension	No	52,448	35.2	4083	7.8	1		
	Yes	95,822	64.4	6007	6.3	0.792	0.760–0.826	<0.0001
Diabetes	No	101,044	67.9	7018	6.9	1		
	Yes	46,241	31.1	3035	6.6	0.941	0.901–0.984	0.007
Anaemia	No	140,813	94.6	9470	6.7	1		
	Yes	7840	5.3	657	8.4	1.269	1.168–1.378	<0.0001
Renal failure	No	139,807	93.9	9191	6.6	1		
	Yes	9035	6.1	947	10.5	1.664	1.551–1.786	<0.0001
Atrialfibrillation	No	11,126	75.0	5746	5.2	1		
	Yes	37,050	25.0	4310	11.6	2.414	2.316–2.516	<0.0001
Dyslipidaemia	No	95,200	63.9	7891	8.3	1		
	Yes	52,857	35.5	2239	4.2	0.489	0.466–0.514	<0.0001
Ischaemic heart disease	No	140,837	94.6	9458	6.7	1		
	Yes	7181	4.8	625	8.7	1.324	1.217–1.441	<0.0001
Readmission	No	141,751	95.2	9154	6.5	1		
	Yes	7140	4.8	987	13.8	2.324	2.165–2.493	<0.0001
SSPF	No	148,231	99.6	10,057	6.8	1		
	Yes	660	0.4	84	12.7	2.004	1.592–2.521	<0.0001
		***n***	**Mean**	**SD**		**Difference of the Means**	**95% CI**	***p***
Age	Survival	138,750	73.34	12.527			−8.872–8.374	
	Death	10,141	81.96	9.510		−8.623	−8.819–8.426	<0.0001
Length of stay	Survival	138,750	7.67	4.501			1.876–2.058	
	Death	10,141	5.71	4.700		1.967	1.873–2.062	<0.0001
NDD	Survival	138,750	6.9224	2.94810			−0.011–0.108	
	Death	10,141	6.8735	3.09802		0.04893	−0.013–0.111	0.1240
NPD	Survival	138,750	3.3534	2.45031			1.099–1.98	
	Death	10,141	2.2044	2.21442		1.14901	1.104–1.194	<0.0001

SSPF: symptoms suggestive of posterior fossa infarction. ORu: odds ratio unadjusted; NDD: number of diagnoses on discharge; NDP: number of procedures on discharge; UA: urgent admission; SA: scheduled admission; COPD: chronic obstructive pulmonary disease; %: percentage from total sample; %*: percentage within the deceased.

**Table 3:** Logistic regression model to predict mortality. Training cohort.

**Logistic Regression**		**Observations**	**=**	**145,400**	
Log likelihood = −32,531.838		Pseudo R^2^	=	0.0975	
Death	OR	SE	P > z	95% CI	
				Lower	Upper
Age	1.069	0.001	0.000	1.067	1.072
Sex	1.202	0.023	0.000	1.149	1.257
Readmission	2.008	0.038	0.000	1.862	2.165
Ischaemic heart disease	1.342	0.046	0.000	1.227	1.467
Arterial hypertension	0.726	0.023	0.000	0.695	0.759
Diabetes mellitus	1.105	0.024	0.000	1.054	1.158
Atrial fibrillation	1.537	0.023	0.000	1.471	1.607
Dyslipidaemia	0.638	0.026	0.000	0.606	0.671
Heart failure	1.518	0.034	0.000	1.421	1.622
SSPF	2.639	0.124	0.000	2.071	3.364

AUC: 0.742, 95% CI: 0.737–0.747; Pearson’s χ2 test: 0.176. SSPF: symptoms suggestive of posterior fossa infarction.

**Table 4:** Risk scores.

Attribute	Points
Age > 76	16
Sex (female)	4
Readmission	9
Ischaemic heart disease	5
Hypertension	−5
Diabetes	1
Atrial fibrillation	7
Dyslipidaemia	−7
Heart failure	6
Symptoms of posterior fossa infarction	14

**Table 5:** Results of the different metrics according to the method of analysis.

	Balanced Accuracy	AUC ROC	Precision	Recall
Logistic regression	0.667	0.742	0.320	0.712
Random forest	0.669	0.726	0.326	0.698
Neural network MLP	0.651	0.651	0.356	0.863

## Data Availability

The data presented in this study are available on request from the corresponding author. The data are not publicly available due to ethical restrictions.
